# Ancient DNA Suggests Single Colonization and Within-Archipelago Diversification of Caribbean Caviomorph Rodents

**DOI:** 10.1093/molbev/msaa189

**Published:** 2020-10-09

**Authors:** Roseina Woods, Ian Barnes, Selina Brace, Samuel T Turvey

**Affiliations:** 1 School of Biological Sciences, Royal Holloway University of London, Egham, United Kingdom; 2 Department of Earth Sciences, Natural History Museum, London, United Kingdom; 3 Institute of Zoology, Zoological Society of London, London, United Kingdom

**Keywords:** Capromyidae, Echimyidae, evolutionary radiation, hutia, island gigantism, Quaternary extinction

## Abstract

Reconstructing the evolutionary history of island biotas is complicated by unusual morphological evolution in insular environments. However, past human-caused extinctions limit the use of molecular analyses to determine origins and affinities of enigmatic island taxa. The Caribbean formerly contained a morphologically diverse assemblage of caviomorph rodents (33 species in 19 genera), ranging from ∼0.1 to 200 kg and traditionally classified into three higher-order taxa (Capromyidae/Capromyinae, Heteropsomyinae, and Heptaxodontidae). Few species survive today, and the evolutionary affinities of living and extinct Caribbean caviomorphs to each other and to mainland taxa are unclear: Are they monophyletic, polyphyletic, or paraphyletic? We use ancient DNA techniques to present the first genetic data for extinct heteropsomyines and heptaxodontids, as well as for several extinct capromyids, and demonstrate through analysis of mitogenomic and nuclear data sets that all sampled Caribbean caviomorphs represent a well-supported monophyletic group. The remarkable morphological and ecological variation observed across living and extinct caviomorphs from Cuba, Hispaniola, Jamaica, Puerto Rico, and other islands was generated through within-archipelago evolutionary radiation following a single Early Miocene overwater colonization. This evolutionary pattern contrasts with the origination of diversity in many other Caribbean groups. All living and extinct Caribbean caviomorphs comprise a single biologically remarkable subfamily (Capromyinae) within the morphologically conservative living Neotropical family Echimyidae. Caribbean caviomorphs represent an important new example of insular mammalian adaptive radiation, where taxa retaining “ancestral-type” characteristics coexisted alongside taxa occupying novel island niches. Diversification was associated with the greatest insular body mass increase recorded in rodents and possibly the greatest for any mammal lineage.

## Introduction

Islands have been considered as “natural laboratories” for understanding evolutionary patterns and dynamics since the 19th century (Wallace 1880; Ricklefs and Bermingham 2008). Determining the processes that generate the distinct biological diversity and morphological disparity of insular biotas constitutes a key aspect of evolutionary research. This diversity can result from adaptive radiation of a founder population into multiple unoccupied niches, or colonization by multiple founders (Schluter 2000; Yoder et al. 2010). Differentiating between these processes is complicated by morphological evolution in unique insular ecological conditions, which frequently generates morphotypes differing markedly from related continental taxa (van der Geer et al. 2010). Molecular analyses are therefore often necessary to determine the evolutionary histories of morphologically unusual island taxa. However, these biotas are often extremely vulnerable to human-caused extinction, and most island systems have experienced considerable biodiversity loss during the Holocene (Turvey 2009), reducing availability of samples for phylogenetic analysis and hindering reconstruction of the evolution of human-disrupted biotas.

The insular Caribbean (Greater and Lesser Antilles and Bahama Archipelago) is a geologically complex oceanic-type island group that represents an important study system for investigating evolutionary patterns and processes (Ricklefs and Bermingham 2008). It is also one of the few noncontinental shelf island groups colonized by multiple nonvolant mammal lineages, and until recently contained a series of island-endemic terrestrial mammal faunas (Woods and Sergile 2001; Turvey 2009; Cooke et al. 2017). Nonvolant mammal faunas in the Greater Antilles, Bahama Archipelago, and northern Lesser Antilles were dominated in species richness and morphological disparity by caviomorph rodents. These taxa occupied numerous terrestrial and arboreal niches, ranging from **∼**0.1 kg to almost 200 kg (Eisenberg 1978; Biknevicius et al. 1993; Turvey and Fritz 2011) and possibly rivaling the largest known rodents in size (Millien and Bovy 2010).

**Fig. 1.**
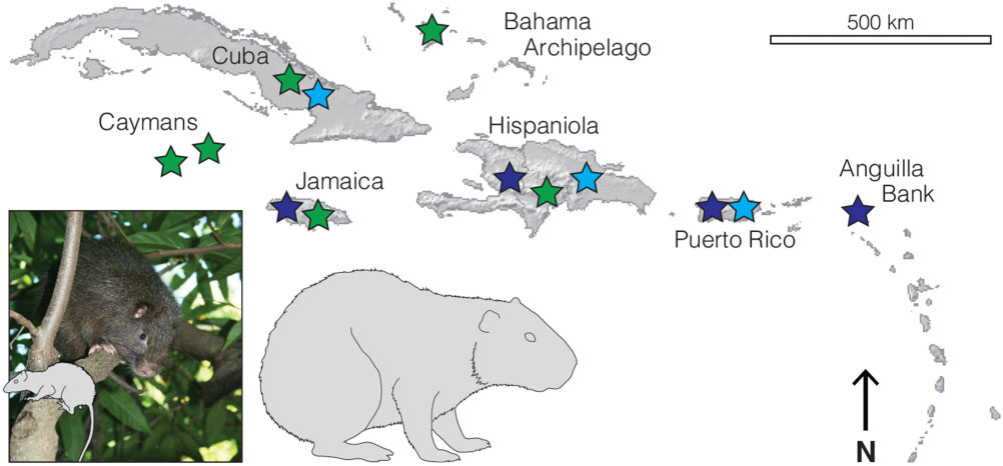
Diversity and distribution of Caribbean caviomorphs. Silhouettes of Hispaniolan spiny rat (*Brotomys voratus*) and Puerto Rican giant hutia (*Elasmodontomys obliquus*) to scale against living Hispaniolan hutia (*Plagiodontia aedium*), and distribution of Capromyidae (green), Heteropsomyinae (pale blue), and Heptaxodontidae (dark blue). Photograph copyright José Nuñez-Miño/The Last Survivors project. Figures appear online in colour.

The remarkable diversity of Caribbean caviomorphs was “almost as great as the diversity of all the remaining South American hystricognaths” (Woods 1982, p. 390). These rodents are usually classified in three higher-order taxa (fig. 1). Caribbean spiny rats are grouped as subfamily Heteropsomyinae within the Echimyidae, the most species-rich caviomorph family (Carvalho and Salles 2004; Burgin et al. 2018). Hutias have usually been assigned to the endemic Caribbean family Capromyidae (McKenna and Bell 1997; Woods et al. 2001; Woods and Kilpatrick 2005). Giant hutias or platetooth hutias are generally much larger-bodied rodents usually placed in another endemic family, Heptaxodontidae (Woods et al. 2001; MacPhee and Flemming 2003; MacPhee 2011). These three groups are differentiated primarily by cheektooth morphology (Woods 1989; MacPhee and Flemming 2003).

Some heptaxodontids probably became extinct before regional human arrival (McFarlane et al. 1998; Morgan and Wilkins 2003). The insular Caribbean also experienced more mammal extinctions than any other global region during the Holocene, probably associated with hunting, landscape transformation, and invasive mammal introduction by Amerindian and subsequent European colonists from **∼**6,000 years ago onward (MacPhee 2009; Turvey 2009; Cooke et al. 2017). From a late Quaternary fauna comprising 33 currently recognized endemic species in 19 genera (Silva Taboada et al. 2007; Turvey and Fritz 2011; Hansford et al. 2012), all Caribbean heteropsomyines and heptaxodontids are now extinct, and only 11 capromyid species in five genera probably survive, most of which are threatened with extinction (Turvey et al. 2017).

Genetic studies of extant taxa reveal that capromyids are nested within the echimyid radiation (Leite and Patton 2002; Galewski et al. 2005; Fabre et al. 2014; Fabre, Upham, et al. 2016; Upham and Borroto-Páez 2017), suggesting this family should be reinterpreted as a subfamily of Echimyidae, as previously suggested by Ellerman (1940). Densely sampled mitogenomic analysis and a recent study using 500 nuclear genes suggest that capromyids are sister to the extant echimyid *Carterodon* (Fabre, Upham, et al. 2016; Courcelle et al. 2019). However, the evolutionary affinities of extinct Caribbean caviomorphs to each other, to capromyids, or to mainland Neotropical caviomorphs are unclear. Heteropsomyines and heptaxodontids are known only from Quaternary material, with some taxa known only from limited material that hinders straightforward morphological comparisons, and with morphology-based classification compromised by widespread convergence of dental characters across caviomorphs (MacPhee 2011; Candela and Rasia 2012; Boivin and Marivaux 2020) and widespread body size change in island taxa (van der Geer et al. 2010).

Caribbean taxa have been suggested to represent either multiple colonizations, with the morphologically more derived capromyids and heptaxodontids possibly representing older colonization(s), or alternately a monophyletic group representing a single colonization (Woods 1982, 1989; McKenna and Bell 1997; Woods et al. 2001; Carvalho and Salles 2004; Woods and Kilpatrick 2005; MacPhee 2011). Timing of caviomorph arrival in the Caribbean is also uncertain because the region’s pre-Pleistocene fossil record is extremely limited; rodent incisors with caviomorph-type enamel microstructure are known from the Oligocene of Puerto Rico (Vélez-Juarbe et al. 2014), and fossils referred to an extinct capromyid genus (*Zazamys*) are known from the Miocene of Cuba (MacPhee et al. 2003). Genetic data have so far been unavailable for extinct Caribbean caviomorphs, and ancient DNA (aDNA) analysis of Caribbean samples remains challenging due to unfavorable conditions for ancient biomolecule preservation in tropical environments (Gutiérrez-García et al. 2014; Brace et al. 2015).

Are Caribbean caviomorph taxa monophyletic (all species result from a single overwater dispersal), polyphyletic (species result from multiple overwater dispersals by different groups), or paraphyletic (one or more named families or subfamilies contain other named family-level or subfamily-level clades)? New efforts to understand the processes that generated the remarkable morphological diversity of the Caribbean’s enigmatic extinct caviomorphs would provide wider insights into the dynamics of insular evolution, the region’s biogeographic history, and the extent to which adaptive radiations can generate novelty in mammalian lineages. As morphology-based studies have been unable to provide robust resolution of Caribbean caviomorph relationships, molecular analysis of extinct species is necessary to reconstruct this evolutionary radiation. Here, we present the first genetic data from late Quaternary subfossil samples of extinct heteropsomyines and heptaxodontids, and from historical and subfossil samples of several extinct capromyids. We use these data to test between previous hypotheses about the group’s higher-order affinities and evolutionary history, and determine the pattern and timing of their diversification using molecular phylogenetic techniques.

## Results

We sampled 24 specimens of extinct Caribbean caviomorphs, representing two heteropsomyines (*Boromys offella*, three specimens; *Brotomys voratus*, one specimen), one giant heptaxodontid (**∼**10 kg *Elasmodontomys obliquus*, six specimens), and five capromyids (*Geocapromys columbianus*, three specimens; *G. thoracatus*, two specimens; *Hexolobodon phenax*, one specimen; *Isolobodon montanus*, one specimen; and *I. portoricensis*, seven specimens) (table 1). Unaccessioned field-collected material was identified using established morphological criteria for these genera and species (Miller 1916,^ 1922^,^ 1929^; Anthony 1918). For each target species, screening was used to select one specimen that provided the highest quality and quantity of endogenous DNA for final sequencing. We successfully extracted and sequenced aDNA from several extinct taxa, including all sampled heteropsomyines and heptaxodontids, and two extinct capromyids (both extinct *Geocapromys* species). Unfortunately, aDNA extraction from available samples of *Isolobodon* and *Hexolobodon* was unsuccessful.

**Table 1.** Samples of Extinct Caribbean Caviomorphs Used in aDNA Analysis.

Species	Island	Site	Material	No. of Samples	Source	Collection Details
1. Heteropsomyinae						
*Boromys offella*	Cuba	Las Obas	Bone (zooarchaeological)	3	PMYU (210009, 2 specimens; 210203)	Colten et al. (2009)
*Brotomys voratus*	Hispaniola	Cueva de la Línea, Samaná Bay, Dominican Republic (19°04′39.5″N, 69°27′57.7″W)	Bone (subfossil)	1	Field collected by STT	—
2. Capromyidae						
*Geocapromys columbianus*	Cuba	Las Obas	Bone (zooarchaeological)	3	PMYU (210020, 210033, 210203)	Colten et al. (2009)
*Geocapromys thoracatus*	Little Swan Island	—	Tissue (museum skin)	2	EXEMS (28/1939/4, 1/1940/1)	Tonge (2014)
*Hexolobodon phenax*	Hispaniola	Cueva de Lelo (San Gabriel Cave), Samaná Bay, Dominican Republic (19°05′19.8″N, 69°30′42.3″W)	Tooth (subfossil)	1	Field collected by STT	—
*Isolobodon montanus*	Hispaniola	Unnamed cave, Parque del Este, Dominican Republic (18°21′43.1″N, 68°37′20.9″W)	Bone (subfossil)	1	Field collected by STT	—
*Isolobodon portoricensis*	Hispaniola	Unnamed cave, Parque del Este, Dominican Republic (18°21′43.1″N, 68°37′20.9″W)	Tooth (subfossil)	1	Field collected by STT	—
*Isolobodon portoricensis*	Guana Island (British Virgin Islands)	Unnamed cave (18°28′26″N, 64°34′15″W)	Tooth (zooarchaeological)	2	Field collected by STT	Lazell (2005)
*Isolobodon portoricensis*	Puerto Rico	Cueva de la Vaca (18°20′43.3″N, 66°27′05.6″W)	Tooth, bone (subfossil)	4	Field collected by STT	Turvey et al. (2007)
3. Heptaxodontidae						
*Elasmodontomys obliquus*	Puerto Rico	Cueva de la Vaca (18°20′43.3″N, 66°27′05.6″W)	Tooth, bone (subfossil)	6	Field collected by STT	Turvey et al. (2007)

Note.—EXEMS, Royal Albert Memorial Museum & Art Gallery, Exeter, UK; PMYU, Peabody Museum of Natural History, Yale University; STT, field collected by senior author.

We recovered whole or partial mitochondrial genome data and five nuclear genes (GHR, RAG1, RBP3, apoB, and vWF) for each species, which were included in phylogenetic analysis (supplementary table S1, Supplementary Material online; GenBank accession numbers MN304800–MN304814). The final length of the whole mitochondrial genome alignment was 15,259 bp and the final length of the concatenated nuclear gene alignment used for analysis was 5,115 bp, but both contained missing data for some taxa (supplementary table S1, Supplementary Material online). Average coverage varied between 2.33 and 63.14**×** for mitochondrial genomes and 0.43 and 20.71**×**for nuclear genes (table 2 and supplementary table S2, Supplementary Material online). We aligned sequence data for extinct species with existing sequence data for 47 extant caviomorphs, including six extant capromyids representing all surviving genera (*Capromys pilorides*, *Geocapromys brownii*, *G. ingrahami*, *Mesocapromys melanurus*, *Mysateles prehensilis*, and *Plagiodontia aedium*).

**Table 2.** Final Coverages of Mitochondrial Genomes and Nuclear Genes Sequenced from Extinct Caribbean Caviomorphs.

Species	Gene	Average Coverage
*Brotomys offella*	Mitochondrial genome	4.84
	vWF	1.64
	RAG1	1.04
	RBP3	1.19
	apoB	0.43
	GHR	0.50
*Brotomys voratus*	Mitochondrial genome	16.69
	vWF	10.50
	RAG1	5.43
	RBP3	8.64
	apoB	3.23
	GHR	2.16
*Elasmodontomys obliquus*	Mitochondrial genome	4.89
	vWF	20.71
	RAG1	14.15
	RBP3	17.90
	apoB	5.91
	GHR	10.72
*Geocapromys columbianus*	Mitochondrial genome	2.33
	vWF	6.95
	RAG1	5.03
	RBP3	8.02
	apoB	0.82
	GHR	3.87
*Geocapromys thoracatus*	Mitochondrial genome	63.14
	vWF	1.85
	RAG1	2.92
	RBP3	1.31
	apoB	3.76
	GHR	1.92

**Fig. 2.**
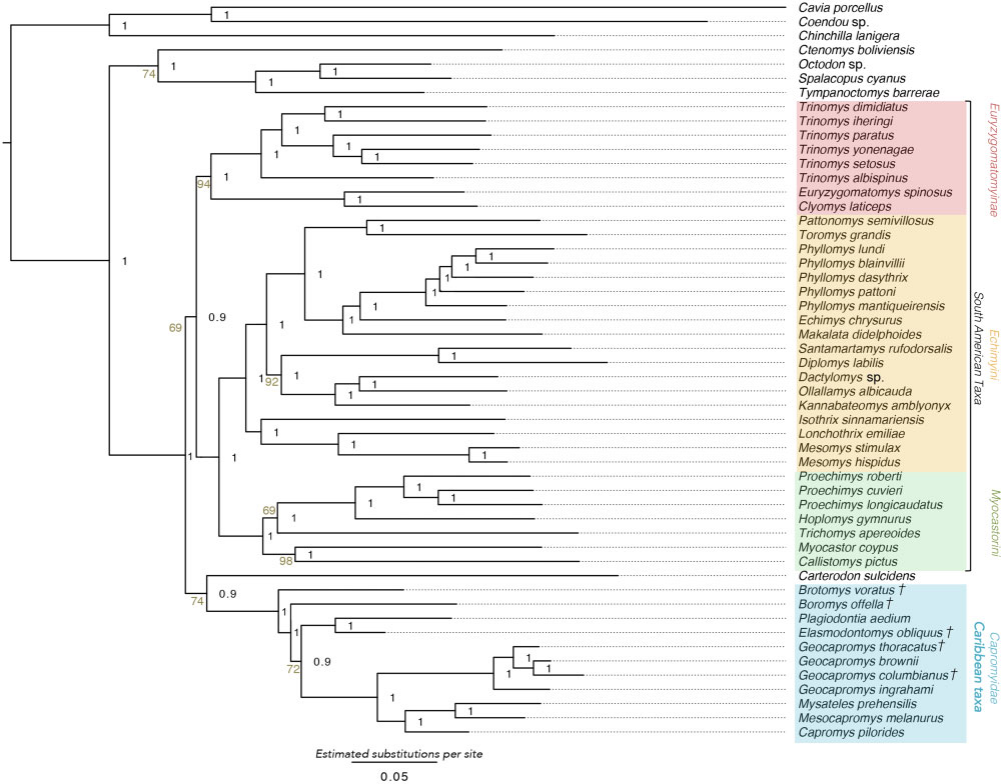
Bayesian phylogeny showing relationships of Caribbean caviomorphs within Neotropical caviomorph radiation. Main clades are color coded and named. Extinct species are indicated with crosses. Node values represent posterior probabilities. Scale indicates nucleotide substitutions per site. Figures appear online in colour.

**Fig. 3.**
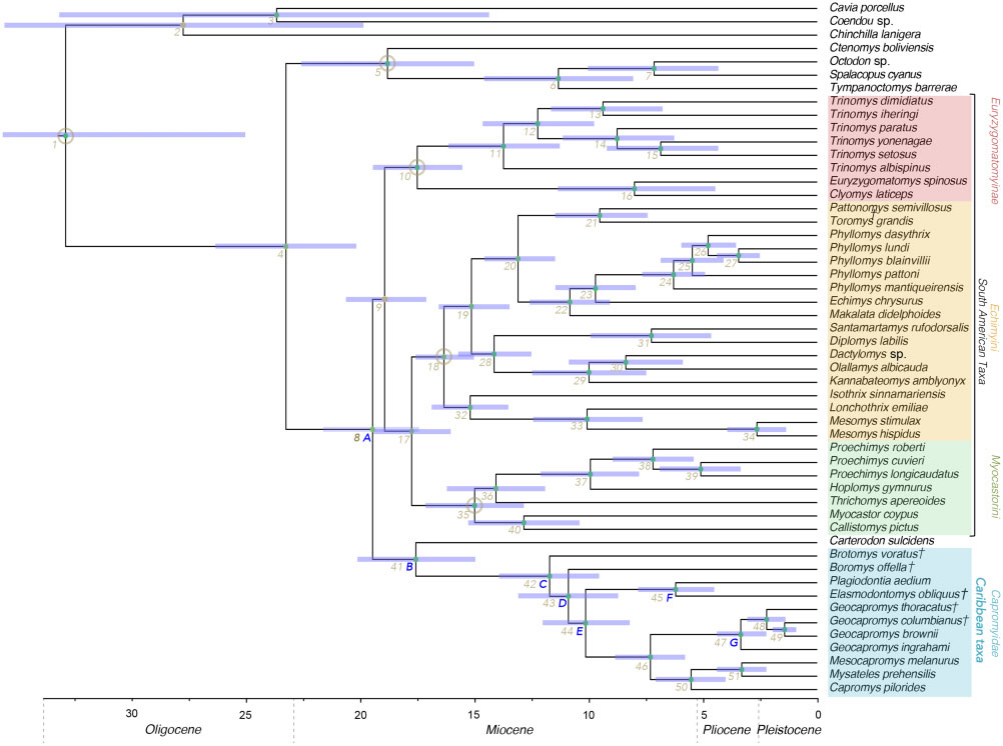
Time-calibrated phylogeny showing estimated divergence dates for Caribbean caviomorphs and mainland Neotropical caviomorphs. Main clades are color coded and named. Extinct species are indicated with crosses. Nodes used for fossil calibration are circled. Node colors represent posterior probabilities (yellow < 1 and green = 1); no posterior probabilities were <0.9. Numbered nodes include 95% error bar estimates for divergence dates. Figures appear online in colour.

Bayesian and Maximum Likelihood analyses were fully congruent and recover all Caribbean caviomorphs included in this study as a monophyletic group, which is sister to the extant mainland echimyid *Carterodon* (Bayesian phylogeny, fig. 2; divergence-dated phylogeny, fig. 3; Maximum Likelihood phylogeny not shown). Time-calibrated Bayesian analysis excluding extinct taxa recovered congruent topology to our primary phylogeny, although support for the placement of *Carterodon* was low (posterior probability <0.5) (supplementary fig. S1, Supplementary Material online). Coalescent-based species tree estimation also recovered a tree topology that was congruent with our Maximum Likelihood and Bayesian trees (supplementary fig. S2, Supplementary Material online), with a normalized quartet score of 0.8 (i.e., 80% of quartets in our gene trees are present within the coalescent-based species tree).

*Brotomys* and *Boromys* show the earliest divergences within the Caribbean clade (nodes 42–43); they are not sister taxa within a distinct heteropsomyine clade but instead represent stem-group taxa that diverge successively at the base of the Caribbean radiation, making Heteropsomyinae paraphyletic with respect to other Caribbean caviomorphs. Other sampled Caribbean taxa fall into two crown-group clades, with the extinct heptaxodontid *Elasmodontomys* nested within the capromyid radiation. One crown-group clade contains capromyids and heptaxodontids from the eastern Greater Antillean islands of Hispaniola and Puerto Rico (*Plagiodontia*, *Elasmodontomys*), and the other contains capromyids from the western Greater Antillean islands of Cuba, Jamaica, Bahamas, the Caymans, and Little Swan Island (*Capromys*, *Geocapromys*, *Mesocapromys*, and *Mysateles*).

Our Bayesian, Maximum Likelihood, and divergence-dated phylogenies all included placement of the *Carterodon* + Caribbean caviomorph clade as sister to all other echimyids (figs. 2 and 3, node 8). Support for placement of this clade is high (posterior probability = 1). Divergence times of all higher-order Caribbean lineages (above the genus level) are estimated to have occurred during the Miocene (supplementary table S3, Supplementary Material online). Divergence between Caribbean caviomorphs and *Carterodon* dates to 18.08 Ma (node 41: 95% highest probability density [HPD], 9.9–21.7 Ma), and the earliest divergence within the Caribbean caviomorphs (between *Brotomys* and the remaining heteropsomyine–capromyid–heptaxodontid clade) dates to 12.05 Ma (node 42: 95% HPD, 7.1–15.1 Ma). The primary divergence of crown-group capromyids + heptaxodontids into eastern and western Greater Antillean clades dates to 10.42 Ma (node 44: 95% HPD, 6.7–14.1 Ma), and divergence between *Elasmodontomys* and *Plagiodontia* dates to 6.37 Ma (node 45: 95% HPD, 3.4–8.8 Ma).

## Discussion

In this study, we were able to overcome the persistent problem of limited DNA preservation in ancient samples from tropical environments and generated the first aDNA sequence data for multiple extinct caviomorph rodents from the insular Caribbean, a globally important hotspot of evolutionary innovation and biodiversity loss. In particular, we report the first mitogenomic data for two ecologically and evolutionarily significant extinct Quaternary mammal groups, the Heteropsomyinae and Heptaxodontidae. Phylogenetic analysis of these data provides important insights into the affinities, evolutionary history, and biogeographic origins of a major mammalian radiation that cannot be understood with modern-day samples, and yields additional insights into dynamics of insular evolution and acquisition of evolutionary novelty in island lineages.

### Colonization History of Caribbean Caviomorphs

Previous morphology-based studies have generated contrasting hypotheses about possible evolutionary affinities of extant and extinct Caribbean caviomorphs. Several authors have interpreted these ecologically and morphologically divergent taxa as a polyphyletic assemblage derived from different Neotropical source populations through multiple colonizations (McKenna and Bell 1997; MacPhee and Flemming 2003; Carvalho and Salles 2004; MacPhee 2011). Heteropsomyines and heptaxodontids have both been interpreted as most closely related to different extinct or extant mainland Neotropical caviomorphs (MacPhee and Flemming 2003; Carvalho and Salles 2004; MacPhee 2011). Heptaxodontids have been variously assigned to three of the four caviomorph superfamilies (Cavioidea, Chinchilloidea, and Octodontoidea), and different heptaxodontid genera have been referred to different higher-order taxa (Kraglievich 1926; Simpson 1945; Landry 1957; Ray 1964,^ 1965^; Woods and Kilpatrick 2005).

However, all taxa that we sampled, including representatives of Capromyidae, Heteropsomyinae, and Heptaxodontidae from Cuba, Hispaniola, Jamaica, Puerto Rico and other islands, represent a single well-supported clade. The remarkable variation observed across these taxa in important ecological parameters such as body size and trophic niche (Eisenberg 1978; Turvey et al. 2006; Cooke and Crowley 2018) was therefore generated through within-archipelago adaptive radiation following a single colonization, in contrast to the origination of diversity in many other Caribbean land mammal groups including oryzomyine rice rats (Brace et al. 2015) and primates (Woods et al. 2018). Although we were only able to include one heptaxodontid in our molecular analyses, our phylogenetic conclusions instead support the morphology-based hypothesis proposed by Allen (1918), Ray (1964,^ 1965^), Woods (1982,^ 1989^), and Woods et al. (2001), who suggested some or all Caribbean caviomorphs were monophyletic, with capromyids derived from heteropsomyines, and heptaxodontids derived from a capromyid ancestor on Hispaniola. More specifically, the Hispaniolan capromyid *Plagiodontia* and the heptaxodontid *Elasmodontomys* were sometimes recovered as sister taxa in cladistic analysis based on morphological (craniodental) characters by Woods et al. (2001) depending upon choice of outgroup in this previous study, matching the phylogenetic placement of these taxa in our study.

Because all sampled Caribbean caviomorphs represent a well-supported clade, our inclusion of extinct species, including two extinct higher-order taxa not previously included in molecular analyses, has little effect on wider tree topology compared with previous studies that sampled only extant taxa. Our estimated divergence of the Caribbean clade during the Early Miocene (early Santacrucian) corresponds closely to estimates for divergence of this clade in previous molecular analyses (18.2 Ma, Galewski et al. 2005; 16.5 Ma, Fabre et al. 2014; 12.7 or 14.4 Ma, Fabre, Upham, et al. 2016; and 15.5 Ma, Upham and Borroto-Páez 2017). The topology recovered for extant Caribbean caviomorphs in our analysis, with a basal split between *Plagiodontia* and other extant capromyids, is also congruent with previous molecular analyses (Fabre et al. 2014; Fabre, Upham, et al. 2016; Upham and Borroto-Páez 2017).

Fabre, Upham, et al. (2016) recovered *Carterodon* as sister to extant capromyids but warned that the deepest relationships within their analysis of extant echimyids were impacted by long branch attraction, in particular involving the long branch of *Carterodon*, and suggested that future sampling of extinct heteropsomyines might overcome this problem. Our analyses again consistently recovered *Carterodon* as sister to Caribbean caviomorphs, and our inclusion of mitogenomic data for *Boromys*, *Brotomys*, and other extinct taxa provides strong support for the *Carterodon* + Caribbean caviomorph clade as being sister to all other echimyids. Analysis conducted with only the extant taxa included in our primary phylogeny recovered the same sister relationship between *Carterodon* and Caribbean caviomorphs, and the recent study by Courcelle et al. (2019), which included additional nuclear markers, is also congruent with our analysis. These studies suggest that additional sampling of extinct taxa and inclusion of slower-evolving nuclear markers, even in isolation, are able to overcome the issues encountered by Fabre, Upham, et al. (2016) and Fabre, Patton, et al. (2016).

Our estimated Early Miocene divergence for the Caribbean caviomorph clade postdates the putative existence of a land-bridge (“GAARlandia”) linking the Greater Antilles with South America near the Eocene–Oligocene boundary **∼**34 Ma (Iturralde-Vinent and MacPhee 1999; Ali 2012). Colonization must therefore have occurred through overwater dispersal, probably via unidirectional ocean currents hypothesized to have carried rafts of vegetation northwest from the mouths of large South American rivers throughout the Cenozoic (Hedges 1996). Our estimated divergence date for this clade is also substantially younger than the oldest reported Caribbean caviomorph fossils, two incisors from Oligocene contexts in Puerto Rico dated to 33.9–28.4 Ma and **∼**27.2–24.7 Ma, which exhibit enamel microstructure characteristic of chinchilloids and cavioids rather than octodontoids (the higher-order group including echimyids and capromyids) (Vélez-Juarbe et al. 2014). As these authors themselves considered, these fossils may therefore represent an earlier colonization event by a different caviomorph lineage that seemingly disappeared before the Quaternary. Some other early Cenozoic Caribbean land mammal groups also became extinct before the Quaternary, possibly due to inundation of subaerial landmasses (Domning et al. 1997; Iturralde-Vinent 2006).

Our divergence estimates also conflict with the estimated age of the other definite Caribbean Neogene caviomorph, *Zazamys veronicae* from the Early Miocene of Cuba. This taxon is known from three cheek teeth referable to an isolobodontine capromyid, from deposits with a minimum age of either 17.5 Ma based on stratigraphy or 14.68 Ma based on strontium isotope analysis (MacPhee et al. 2003). Although we were unable to amplify aDNA from *Isolobodon* to resolve its affinities, isolobodontine capromyids are uncontroversially interpreted as part of the crown-group capromyid radiation that diversified within the Caribbean (Woods et al. 2001). However, both available age estimates for *Zazamys* are older than our estimate for diversification of the crown-group capromyid radiation (95% HPD = 6.7–14.1 Ma), although the strontium age estimate falls within the upper bound of the 95% HPD for divergence of stem-group heteropsomyine lineages exhibiting “echimyid-type” morphology (*Boromys* and *Brotomys* divergences, 95% HPD = 7.1–15.1 Ma). This lack of congruence between fossils and molecules could indicate that our divergence date estimates are too young, that available dates for *Zazamys* are too old, or that *Zazamys* is not really an isolobodontine. The **∼**3 Ma discrepancy between minimum age estimates for *Zazamys* obtained using different geological dating methods (MacPhee et al. 2003) might suggest inaccurate dating of this fossil, and we encourage further investigation into temporal contexts of Cenozoic Caribbean mammal fossils to better constrain evolutionary hypotheses in future studies.

### Within-Archipelago Diversification and Caviomorph Classification

Our results confirm phylogenetic placement of Caribbean caviomorphs within the Neotropical echimyid radiation, as demonstrated by previous molecular studies (Leite and Patton 2002; Galewski et al. 2005; Fabre et al. 2014; Fabre, Upham, et al. 2016; Upham and Borroto-Páez 2017). The entire clade is sister to *Carterodon*, and basal stem-group lineages within the clade also exhibit plesiomorphic “echimyid-type” morphology and have always been referred to Echimyidae in previous taxonomic treatments. Following previous authors, we therefore recommend that the Caribbean clade is classified as a lower-order taxon within Echimyidae and is not recognized as a distinct mammal family, to prevent Echimyidae from comprising a paraphyletic assemblage. Previous classifications of Caribbean caviomorphs as representing multiple distinct mammal families must therefore also be revised, and we propose that the entire sampled Caribbean radiation should be recognized as a single (albeit biologically remarkable) subfamily within Echimyidae. The oldest available name to describe this clade is Capromyinae Smith 1842.

The Cuban and Hispaniolan heteropsomyines *Boromys* and *Brotomys* are morphologically very similar small-bodied taxa and have even been assigned to the same genus by some authors (Rímoli 1976; Woods et al. 2001). However, our analysis reveals that this similarity merely represents retention of “ancestral echimyid” characteristics rather than recent common ancestry, and these genera represent a paraphyletic assemblage or “spiny rat grade.” Although we were unable to sample the type genus *Heteropsomys* from Puerto Rico and so cannot assess its affinities to Cuban or Hispaniolan heteropsomyines, our results demonstrate that the higher-order taxon Heteropsomyinae Anthony 1917 does not represent a monophyletic group and should therefore no longer be used as a taxonomic category.

Taxa sampled in this study from the crown-group “capromyid-heptaxodontid” clade show a basal split **∼**10 Ma between eastern and western Caribbean taxa, with eastern lineages (Hispaniola and Puerto Rico) diverging **∼**6 Ma. We propose the western clade, comprising the sampled genera *Capromys*, *Geocapromys*, *Mesocapromys* and *Mysateles*, is referred to as Capromyini. *Elasmodontomys* is the type genus of Heptaxodontidae (*Heptaxodon* is a junior synonym of *Elasmodontomys*; Ray 1964). We therefore propose the previous family-level ranking Heptaxodontidae Anthony 1917, in the new tribe combination Heptaxodontini, is used to refer to the eastern clade, comprising the sampled genera *Plagiodontia* and *Elasmodontomys*. This name has seniority over the other name available for this clade, Plagiodontini Ellerman (1940).

Estimated divergence between Capromyini and Heptaxodontini postdates separation of eastern Cuba and northern Hispaniola 25–20 Ma (Pindell and Barrett 1990; Iturralde-Vinent and MacPhee 1999), indicating that phylogenetic divergence was probably associated with overwater dispersal. The physical connection between Hispaniola and Puerto Rico was severed by formation of the Mona Passage, which may have commenced in the mid-Oligocene **∼**30 Ma but may not have been fully inundated until during or after the Miocene (Iturralde-Vinent and MacPhee 1999; Iturralde-Vinent 2006). Phylogenetic divergence between Hispaniolan and Puerto Rican taxa may thus instead represent a vicariance event.

We recognize that our proposed classification of crown-group Caribbean caviomorph clades may be revised if molecular data become available for other previously recognized Caribbean subfamilies that could not be included in this analysis. Hispaniola’s late Quaternary fauna included hutias that were classified by previous authors in three subfamilies (Plagiodontinae, Isolobodontinae, and Hexolobodontinae; Woods et al. 2001; Borroto-Páez et al. 2005; Woods and Kilpatrick 2005), but we were unable to obtain aDNA data from two of these subfamilies.

It is also possible that different heptaxodontids are convergent on large body size and dental morphology and may not be monophyletic. In particular, the giant Jamaican rodent *Clidomys* is sometimes referred to its own subfamily, of uncertain higher-order taxonomic position (Woods 1989; Woods et al. 2001; Woods and Kilpatrick 2005). Analysis of basicranial morphology of the giant Anguilla Bank rodent *Amblyrhiza* has also suggested no close relationship with *Elasmodontomys* (MacPhee 2011). Indeed, although *Geocapromys* (part of the crown-group radiation) occurs on Jamaica, this island’s Quaternary-modern fauna is biogeographically distinct from other Greater Antillean islands, probably because the deep Cayman Trough acts as a marine barrier (Donnelly 1994); other vertebrate groups known from both Jamaica and elsewhere in the Caribbean (e.g., anoles and rice rats) colonized these regions independently (Hedges and Burnell 1990; Musser and Carleton 2005). It is therefore possible that *Amblyrhiza* and/or *Clidomys* might instead represent a separate overwater colonization of the Caribbean by caviomorphs, such as that evidenced by the Oligocene fossils from Puerto Rico (Vélez-Juarbe et al. 2014). Unfortunately, both taxa probably went extinct before the terminal Pleistocene (McFarlane et al. 1998; Morgan and Wilkins 2003) and available specimens are almost certainly beyond the thermal age limit of DNA preservation, so their phylogenetic position is unlikely to be resolvable using genetic methods.

### Acquisition of Novelty in Island Lineages

Our reconstruction of caviomorph evolutionary history and relationships has important implications for understanding processes responsible for generating diversity in island faunas. The Caribbean caviomorphs represent an important new example of insular mammalian adaptive radiation, where a single colonizing lineage from a relatively morphologically conservative mainland clade (Fabre, Patton, et al. 2016) diversified ecologically and morphologically to occupy a variety of terrestrial and arboreal herbivore niches, and with diversity generated through both within-island and between-island radiation. Some taxa (Caribbean spiny rats) retained “ancestral-type” morphology (Carvalho and Salles 2004), possibly because they remained associated with niches similar to those occupied by mainland echimyids, but these taxa coexisted in Quaternary landscapes alongside caviomorphs occupying novel niches present in insular ecosystems.

Caviomorph diversification in the Caribbean was also associated with increased body mass. Whereas mean body mass of South American echimyids is 0.32 kg (mean of 69 extant species; Jones et al. 2009), and *Boromys* and *Brotomys* were also **∼**0.3–0.4 kg, the largest taxon included in our study (*Elasmodontomys*) is >30 times larger (Turvey and Fritz 2011). This evolutionary radiation may have included taxa >600 times larger if *Amblyrhiza* is also within the Heptaxodontini (Biknevicius et al. 1993). These body mass differences provide a dramatic example of insular gigantism of small-bodied taxa consistent with the well-known “island rule” and represent the greatest increases recorded for any living or extinct rodents, and the greatest for any mammal lineage in the case of *Amblyrhiza* (Lomolino et al. 2013). Our study complements and provides wider spatiotemporal perspective on previous molecular analyses of extant allopatric Caribbean land mammal populations, which show that within-island diversification of hutias and other taxa was associated with regional geological histories and historical barriers to gene flow (Brace et al. 2012; Turvey et al. 2016).

Although Caribbean caviomorphs lack some of the unusual adaptations exhibited in other insular rodents (e.g., carnivory, which evolved independently four times within rodents in the Philippines, Sulawesi, and Sahul; Rowe et al. 2016), they are among the most remarkable, species-rich, and divergent of the many insular rodent adaptive radiations. They display comparable disparity of body forms and much greater range of body sizes to other insular radiations, such as Madagascar’s nesomyines and the “old endemic” murines of Sahul (Rowe et al. 2008; Goodman and Monadjem 2017). However, in contrast to many other diverse tropical insular rodent faunas (e.g., Philippines and Wallacea; Jansa et al. 2006; Fabre et al. 2013), the ecological and morphological disparity observed in Caribbean taxa sampled in this study was generated from a single colonization.

The Caribbean caviomorphs therefore constitute an important but relatively overlooked mammal group with great potential for investigating key questions in evolutionary biology and biogeography that have previously been explored with other insular mammals, including the relationship between speciation and geographic factors such as island size and environmental heterogeneity (Justiniano et al. 2015; Heaney et al. 2018). Future studies to clarify the trophic niches and precise distributions of extinct Quaternary taxa would contribute further to understanding the dynamics of the caviomorph radiation, and we hope that the phylogenetic affinities of additional extinct taxa will be clarified using ancient biomolecular methods. Recognition of this remarkable, but largely extinct, mammalian adaptive radiation also highlights the need to improve conservation actions to protect the last surviving representatives of the threatened endemic Caribbean rodent fauna (Turvey et al. 2017).

## Materials and Methods

We carried out extractions in a dedicated aDNA laboratory (Natural History Museum, London), in a separate location to post-polymerase chain reaction analysis. Bone powder (approximate mean weight 1.8 g) from whole or fragmented teeth, skulls, and/or diagnostic skeletal elements was sampled using a hand-held Dremel drill with a 2–3-mm drill bit. To prevent contamination, the surface of the area of bone sampled was removed and discarded, drill bits were changed between samples, and equipment was sterilized with bleach and UV-treated before and after use. Extraction protocol included use of proteinase K for bone digestion and silica spin columns for DNA purification, and followed protocols in Brace et al. (2019).

We built single-index double-stranded DNA libraries following protocols in Meyer and Kircher (2010). Negative extraction and library-build controls were included during each process. We first screened libraries for endogenous DNA using Illumina Next-Generation Sequencing platforms (initially using MiSeq, and then using NextSeq 500). To increase sequencing depth of targeted areas, we used hybridization-capture enrichment before sequencing using the Illumina NextSeq 500 (Enk et al. 2014). We used capture-enrichment kits (MYcroarry, Arbor Biosciences) and designed baits from whole mitochondrial genomes (length 16,816 bp) and five nuclear genes (apoB, length: 1,155 bp; RAG1, length: 1,072 bp; vWF, length 1,150 bp; RBP3, length 1,245 bp; and GHR, length 798 bp) available for echimyids on GenBank (supplementary table S4, Supplementary Material online).

We conducted postsequencing data processing using CLC Workbench v.8 (CLC Bio-Qiagen, Aarhus, Denmark), where reads were paired, merged, and trimmed of adapters using default settings. In order to assess contamination, a Basic Local Alignment Search Tool (BLAST) search (Altschul et al. 1997) was conducted on both raw and final consensus sequence data. To limit deamination-based error, end-bases of reads were removed during postsequencing data processing. Average coverage was low for some genes for some species, particularly for extinct species; this can be accounted for by the low quantity of endogenous DNA in ancient samples and by the fact that some species reference sequence data were not available for mapping. Our choice of multiple molecular markers, including both whole mitochondrial genome and multiple nuclear genes, was made in anticipation of low coverage and missing data associated with these issues. To account for potential mapping ascertainment bias during sequence assembly for extinct species with no close reference sequences, we applied an iterative mapping process following Westbury et al. (2017). We mapped reads to four different reference sequences for the whole mitochondrial genome and each nuclear gene as part of the iterative mapping process outlined above (supplementary table S5, Supplementary Material online). We used all four reference sequences in six separate read mappings, using increasingly strict parameters (supplementary tables S6 and S7, Supplementary Material online). We extracted multiple consensus sequences for each sample, one for each reference sequence and for each parameter from read mappings. We then aligned consensus sequences derived from the same sample to different reference sequences with the same mapping parameter, removed variations between sequences using Gblocks v.0.9 (Talavera and Castresana 2007), and extracted a single consensus sequence. We further aligned these mapping parameter consensus sequences with each other before extracting a final consensus sequence for each sample. Alignments were then translated to check for amino acid changes and unexpected stop codons.

We aligned these sequence data with existing sequence data for 47 extant caviomorphs (representing all extant capromyid genera, all extant echimyid subfamilies, and all caviomorph superfamilies) (supplementary table S8, Supplementary Material online), using ClustalW (Larkin et al. 2007) implemented in Geneious v.8.0.5 (Kearse et al. 2012). We concatenated resulting alignments for mitochondrial genomes and nuclear genes using Seaview v.4 (Gouy et al. 2010).

We implemented Maximum Likelihood and Bayesian methods to estimate phylogenetic relationships, and chose DNA substitution models for the partitioned data set using PartitionFinder (Lanfear et al. 2012) (supplementary table S9, Supplementary Material online). We generated a Maximum Likelihood phylogeny and bootstrap support values using RAxML v.8 (Stamatakis 2014), implemented in CIPRES Science Gateway v.3.3 (Miller et al. 2010). We generated Bayesian phylogenies using MrBayes (Ronquist et al. 2012), with four chains (three heated and one cold) run for 1** × **10^6^ generations, sampling every 1** × **10^3^ generations with a burn-in of 250 trees. Missing data were not removed from Bayesian analysis but were coded as gap characters that did not contribute any probability to the likelihood for that branch and site. Estimated sample size was >200, and potential scale reduction factor, used to assess chain convergence, was close to 1. To assess incomplete lineage sorting, we estimated a coalescent-based species tree using ASTRAL-II v4.10.11 (Mirarab and Warnow 2015; Sayyari and Mirarab 2016).

We generated a divergence-dated phylogeny, estimated under an uncorrelated relaxed lognormal molecular clock (Drummond et al. 2006; coefficient of variation of rates for this clock model = 0.3) using BEAST v.1.8.3 (Drummond et al. 2012), with best-fit evolutionary models chosen using PartitionFinder; missing data were again treated as gap characters rather than being removed. We ran two speciation models (Yule and birth–death models) for comparison; both generated identical topology. We tested model selection further using Path Sampling and Stepping-Stone Sampling marginal likelihood estimation. Log Bayes factors from marginal likelihood estimation analysis indicated that the Yule speciation model was the best fit (Bayes factor > 1) (Baele et al. 2013). We set prior distributions of five nodes using fossil calibration points (supplementary table S10, Supplementary Material online) and left all other priors as default values in BEAUti v.1.8.3 (Drummond et al. 2012). Analysis was run for 25 million generations, sampling every 1,000 generations. We examined convergence and effective sample size for all parameters using Tracer v.1.6.0 (Rambaut et al. 2018) after a burn-in of 25%. We assessed convergence further through additional Markov chain Monte Carlo analysis, which we compared with the output of the initial analysis.

## Supplementary Material

Supplementary data are available at *Molecular Biology and Evolution* online.

## Supplementary Material

msaa189_Supplementary_Data
