# Supplementary material for: Ancient DNA Suggests Single Colonization and Within-Archipelago Diversification of Caribbean Caviomorph Rodents
Source: Mol Biol Evol. 2020 Oct 9;38(1):84–95. doi: 10.1093/molbev/msaa189 (PMC7783164; doi:10.1093/molbev/msaa189)
Supplement: msaa189_Supplementary_Data [file msaa189_supplementary_data.pdf]

Table S1. Sequencing results, including pre-target capture enrichment screening results and post-capture results. Endogenous content and enrichment factor estimated using mapping results from the whole mitochondrial genome reference sequence. Sequence data used in final analyses highlighted in green.

Species	Samples in final analysis	Pre-capture					Post-capture					Enrichment factor	% incr/ decr reads mapped
		Paired merged reads	Average coverage	Reads mapped	Reads mapped mtgenome	Estim. endogen. content	Paired merged reads	Average coverage	Reads mapped	Reads mapped mtgenome	Estim. endogen. content		
G. thoracatus	1	2,709,500	72.3 (mtgen)<br>0.17 (vwF)<br>0.34 (RAG1)<br>0.2 (RBP3)<br>0.39 (apoB)<br>0.38 (GHR)	16051 (mtgen)<br>5 (vwF)<br>7 (RAG1)<br>4 (RBP3)<br>9 (apoB)<br>8 (GHR)	12,927	0.477	5,405,344	63.14 (mtgen)<br>1.85 (vwF)<br>2.92 (RAG1)<br>1.31 (RBP3)<br>3.76 (apoB)<br>1.92 (GHR)	12927 (mtgen)<br>29 (vwF)<br>46 (RAG1)<br>23 (RBP3)<br>54 (apoB)<br>23 (GHR)	16,051	0.297	-61%	19%
E. obliquus	1	2,317,525	0.4 (mtgen)<br>0 (vwF)<br>0 (RAG1)<br>0 (RBP3)<br>0 (apoB)<br>0 (GHR)	6.18E-03 (mtgen)<br>0 (vwF)<br>0 (RAG1)<br>0 (RBP3)<br>0 (apoB)<br>0 (GHR)	6.18E-03	2.67E-07	4,426,535	0.45 (mtgen)<br>2.88 (vwF)<br>1.47 (RAG1)<br>2.9 (RBP3)<br>0.3 (apoB)<br>0.86 (GHR)	261 (mtgen)<br>146 (vwF)<br>71 (RAG1)<br>157 (RBP3)<br>16 (apoB)<br>31 (GHR)	261	0.006	100%	100%
		13,172,455	4.13 (mtgen)<br>2.07 (vwF)<br>1.41 (RAG1)<br>1.79 (RBP3)<br>0.59 (apoB)<br>1.07 (GHR)	3764 (mtgen)<br>132 (vwF)<br>845 (RAG1)<br>230 (RBP3)<br>378 (apoB)<br>490 (GHR)	3764	0.029	63,079,541	4.89 (mtgen)<br>20.71 (vwF)<br>14.15 (RAG1)<br>17.9 (RBP3)<br>5.91 (apoB)<br>10.72 (GHR)	4135 (mtgen)<br>1324 (vwF)<br>845 (RAG1)<br>1230 (RBP3)<br>378 (apoB)<br>490 (GHR)	4135	0.007	-336%	9%

Species	Samples in final analysis	Pre-capture					Post-capture					Enrichment factor	% incr/ decr reads mapped
		Paired merged reads	Average coverage	Reads mapped	Reads mapped mtgenome	Estim. endogen. content	Paired merged reads	Average coverage	Reads mapped	Reads mapped mtgenome	Estim. endogen. content		
B. voratus	1	1,517,978	7.14 (mtgen)<br>0.20 (vwF)<br>0.18 (RAG1)<br>0.34 (RBP3)<br>0.24 (apoB)<br>0.13 (GHR)	1934 (mtgen)<br>6 (vwF)<br>6 (RAG1)<br>10 (RBP3)<br>4 (apoB)<br>2 (GHR)	1,934	0.127	46,254,515	16.69 (mtgen)<br>10.5 (vwF)<br>5.43 (RAG1)<br>8.64 (RBP3)<br>3.23 (apoB)<br>2.16 (GHR)	47251 (mtgen)<br>204 (vwF)<br>59 (RAG1)<br>74 (RBP3)<br>100 (apoB)<br>25 (GHR)	47,251	0.102	-25%	96%
		1,252,406	1.36 (mtgen)<br>0.37 (vwF)<br>0.25 (RAG1)<br>0.25 (RBP3)<br>0.13 (apoB)<br>0.11 (GHR)	569 (mtgen)<br>21 (vwF)<br>14 (RAG1)<br>11 (RBP3)<br>8 (apoB)<br>4 (GHR)	569	0.045	44,000,746	1.69 (mtgen)<br>1.92 (vwF)<br>1.72 (RAG1)<br>2.09 (RBP3)<br>0.36 (apoB)<br>0.81 (GHR)	2519 (mtgen)<br>102 (vwF)<br>94 (RAG1)<br>129 (RBP3)<br>22 (apoB)<br>31 (GHR)	2519	0.006	-694%	77%
B. offella	1	2,140,257	0.33 (mtgen)<br>0.05 (vwF)<br>0.09 (RAG1)<br>0.13 (RBP3)<br>0.06 (apoB)<br>0.02 (GHR)	327 (mtgen)<br>2 (vwF)<br>3 (RAG1)<br>6 (RBP3)<br>2 (apoB)<br>1 (GHR)	326	0.015	52,400,401	4.84 (mtgen)<br>1.64 (vwF)<br>1.04 (RAG1)<br>1.19 (RBP3)<br>0.43 (apoB)<br>0.50 (GHR)	1620 (mtgen)<br>104 (vwF)<br>64 (RAG1)<br>86 (RBP3)<br>28 (apoB)<br>23 (GHR)	16,131	0.031	51%	98%
		1,102,436	8.90E-04 (mtgen)<br>0 (vwF)<br>0 (RAG1)<br>0 (RBP3)<br>0 (apoB)<br>0 (GHR)	1 (mtgen)<br>0 (vwF)<br>0 (RAG1)<br>0 (RBP3)<br>0 (apoB)<br>0 (GHR)	1	9.07E-05	—	—	—	—	—	—	—

Species	Samples in final analysis	Pre-capture					Post-capture					Enrichment factor	% incr/ decr reads mapped
		Paired merged reads	Average coverage	Reads mapped	Reads mapped mtgenome	Estim. endogen. content	Paired merged reads	Average coverage	Reads mapped	Reads mapped mtgenome	Estim. endogen. content		
G. columbianus	1	3,762,236	1.56 (mtgen)<br>6.67 (vwF)<br>3.27 (RAG1)<br>6.49 (RBP3)<br>0.86 (apoB)<br>0 (GHR)	1112 (mtgen)<br>447 (vwF)<br>186 (RAG1)<br>410 (RBP3)<br>57 (apoB)<br>0 (GHR)	1112	0.030	78,429,431	2.33 (mtgen)<br>6.95 (vwF)<br>5.03 (RAG1)<br>8.02 (RBP3)<br>0.82 (apoB)<br>3.87 (GHR)	3363 (mtgen)<br>396 (vwF)<br>281 (RAG1)<br>505 (RBP3)<br>51 (apoB)<br>160 (GHR)	3363	0.004	-589%	67%
		3,451,270	0.04 (mtgen)<br>0.69 (vwF)<br>0.10 (RAG1)<br>0.44 (RBP3)<br>0.16 (apoB)<br>0 (GHR)	23 (mtgen)<br>21 (vwF)<br>6 (RAG1)<br>26 (RBP3)<br>4 (apoB)<br>0 (GHR)	23	0.001	40,120,287	0.07 (mtgen)<br>0.37 (vwF)<br>0.11 (RAG1)<br>0.02 (RBP3)<br>0.39 (apoB)<br>0.08 (GHR)	109 (mtgen)<br>39 (vwF)<br>23 (RAG1)<br>2 (RBP3)<br>25 (apoB)<br>3 (GHR)	109	2.00E-04	-145%	79%
I. portoricensis	0	137,018	0.04 (mtgen)<br>0 (vwF)<br>0 (RAG1)<br>0 (RBP3)<br>0 (apoB)<br>0 (GHR)	14 (mtgen)<br>0 (vwF)<br>0 (RAG1)<br>0 (RBP3)<br>0 (apoB)<br>0 (GHR)	14	0.010	—	—	—	—	—	—	—

Table S2. Full details of final coverages of mitochondrial genomes and nuclear genes sequenced from extinct Caribbean caviomorphs.

**Species**	**Gene**	**Missing data (bp)**	**Frequency (%)**	**GC content %**	**Sequence length (bp)**
B. offella	mitochondrial genome	1,721	11.00	35.5	15,912
	vWF	241	37.60	61	809
B. voratus	mitochondrial genome	1,545	8.20	38.6	15,912
	vWF	8	1.40	61.1	809
	RAG1	32	3.00	53.2	1,073
	apoB	32	2.80	39.1	1,151
	GHR	19	2.20	49	842
E. obliquus	mitochondrial genome	1,666	8.40	38.9	15,912
	vWF	23	2.80	59.8	809
	RAG1	31	2.90	53.2	1,073
	RBP3	30	4.00	61.7	753
	apoB	45	3.90	39.4	1,151
	GHR	43	5.10	49.7	842
G. columbianus	mitochondrial genome	5,462	29.50	39.1	15,912
	vWF				
	RAG1	682	63.70	64.5	1,073
	RBP3	148	12.40	63.6	753
	GHR	293	36.10	50.9	842
G. thoracatus	mitochondrial genome	272	1.80	35.8	15,912
	RAG1	0	0.00	52.4	1,073
	RBP3	13	1.00	61.7	753
	apoB	3	0.30	39.3	1,151
	GHR	21	2.60	50.8	842

Table S3. Estimated divergence dates for numbered and lettered nodes in time-calibrated caviomorph phylogeny shown in Figure 3.

Node	Mean estimated divergence date (Mya)	95% HPD (Mya)	Node	Mean estimated divergence date (Mya)	95% HPD (Mya)
1	33.87	16.1, 42.1	***27***	3.52	2.0, 4.9
2	28.58	6.3, 37.7	***28***	14.55	11.7, 17.1
3	24.36	4.2, 35.2	***29***	10.28	7.0, 13.0
4	23.94	21, 27.5	***30***	8.61	5.5, 11.5
5	19.36	7.3, 23.0	***31***	7.46	4.7, 10.3
6	11.65	4.9, 16.1	***32***	15.63	6.9, 12.9
7	7.34	2.9, 11.4	***33***	10.36	7.0, 13.4
8	20.04	18.1, 22.4	***34***	2.70	1.3, 4.2
9	19.49	17.7, 21.7	***35***	15.43	9.2, 18.5
10	18.02	10, 21.2	***36***	14.47	8.1, 17.4
11	14.13	7.6, 17.4	***37***	10.21	5.5, 12.9
12	12.58	6.6, 15.8	***38***	7.39	3.9, 9.9
13	9.64	4.7, 13.1	***39***	5.24	2.6, 7.5
14	9.00	4.7, 13	***40***	13.21	7.4, 16.4
15	7.04	2.9, 9.7	***41***	18.08	9.9, 21.7
16	8.22	4.1, 11.9	***42***	12.05	7.1, 15.1
17	18.27	15.8, 20.3	***43***	11.20	7.1, 15.1
18	16.81	15.8 18.3	***44***	10.42	6.7, 14.1
19	15.57	14.1, 17.6	***45***	6.37	3.4, 8.8
20	13.47	11.5, 15.6	***46***	7.50	6.4, 13.3
21	9.79	6.5, 12.4	***47***	3.43	1.6, 4.6
22	11.00	8.8, 13.4	***48***	2.26	1.2, 3.2
23	9.98	7.5, 12.1	***49***	1.45	0.6, 2.2
24	6.46	4.4, 8.3	***50***	5.67	2.8, 7.6
25	5.61	3.7, 7.4	***51***	3.39	1.4, 4.9
26	4.90	3.1, 6.6			

Table S4. Genes used to generate baits for capture enrichment.

Gene	Length (bp)	NCBI accession	Species
apolipoprotein B (apoB)	1155	JX515310.1	*Proechimys quadruplicatus*
recombination activating gene 1 (RAG1)	1072	EU313333.1	*Proechimys simonsi*
von Willebrand factor (vWF)	1150	AJ849311.1	*Echimys didelphoides*
interphotoreceptor retinoid binding (RBP3)	1245	JF297775.1	*Phyllomys* sp.
growth hormone receptor (GHR)	798	JX515330.1	*Trinomys setosus*
mitochondrial genome	16816	HM544128.1	*Proechimys longicaudatus*

Table S5. Sequence data used in post-sequencing data processing as reference sequences in read mappings.

Gene	Species	NCBI accession number
mitochondrial genome	*Plagiodontia aedium*	KU892771.1
	Mysateles prehensilis	KU892770.1
	Chinchilla lanigera	NC021386.1
	Carterodon sulcidens	KU892752.1
	Capromys pilorides	KU892766.1
	Proechimys longicaudatus	NC020657.1
	Geocapromys brownii	KU892767.1
	Mesocapromys melanurus	KU892769.1
GHR	*Trinomys paratus*	JX515330.1
	Capromys pilorides	AF433950.1
	Geocapromys brownii	KM013984.1
	Geocapromys ingrahami	KM013985.1
	Mesocapromys auritus	KJ742633.1
	Mesocapromys angelcabrerai	KJ742632.1
	Mysateles prehensilis	KJ742634.1
RAG1	*Proechimys simonsi*	EU313333.1
	Capromys pilorides	JN633628.1
	Geocapromys ingrahami	KM013990.1
	Geocapromys brownii	KM013989.1
	Mesocapromys angelcabrerai	KJ742694.1
	Mesocapromys auritus	KJ742693.1
	Mesocapromys melanurus	KJ742691.1
	Mysateles prehensilis	KJ742696.1
RBP3	*Phyllomys* sp.	JF297775.1
	Capromys pilorides	KM013993.1
	Geocapromys ingrahami	KM013995.1
apoB	*Proechimys quadruplicatus*	JX515310.1
vWF	*Echimys didelphoides*	AJ849311.1
	Capromys pilorides	AJ251142.1
	Geocapromys ingrahami	KM014000.1
	Geocapromys brownii	KM013999.1
	Mesocapromys auritus	KJ742623.1
	Mesocapromys angelcabrerai	KJ742622.1

Table S6. Results of reads-mapping analysis conducted in CLC Workbench v.8 for iterative consensus sequence generation.

Species	Mapping parameters	Reference sequence	Total reads mapped	Average coverage
Geocapromys columbianus	M1	*G. ingrahami*	124	0.21
		M. prehensilis	34	0.06
		P. aedium	25	0.05
		P. longicaudatus	43	0.04
	M	*G. ingrahami*	450	0.55
		M. prehensilis	337	0.75
		P. aedium	357	0.75
		P. longicaudatus	293	0.34
	L	*G. ingrahami*	679	1.78
		M. prehensilis	360	0.7
		P. aedium	560	1.03
		P. longicaudatus	618	1.17
Geocapromys thoracatus	XH	*G. ingrahami*	5987	16.89
		M. prehensilis	1387	3.42
		P. aedium	937	2.16
		P. longicaudatus	432	0.91
	H1	*G. ingrahami*	20573	82.3
		M. prehensilis	3592	10.73
		P. aedium	2260	6.23
		P. longicaudatus	1156	2.82
	H	*G. ingrahami*	30958	131.89
		M. prehensilis	5744	18.55
		P. aedium	3967	12.24
		P. longicaudatus	2559	7.47
	M1	*G. ingrahami*	33131	140.26
		M. prehensilis	6492	21.42
		P. aedium	5226	17.3
		P. longicaudatus	3914	12.67
	M	*G. ingrahami*	34067	143.05
		M. prehensilis	6847	22.43
		P. aedium	5765	19.22
		P. longicaudatus	4877	16.33
	L	*G. ingrahami*	37001	148.4
		M. prehensilis	8653	25.06
		P. aedium	7889	21.96
		P. longicaudatus	7592	19.73
Elasmodontomys obliquus	H	*G. ingrahami*	39	0.05
		M. prehensilis	25	0.04
		P. aedium	52	0.07
		P. longicaudatus	43	0.05

	M1	*G. ingrahami*	149	0.18
		M. prehensilis	123	0.15
		P. aedium	196	0.25
		P. longicaudatus	194	0.22
	M	*G. ingrahami*	345	0.42
		M. prehensilis	292	0.36
		P. aedium	450	0.56
		P. longicaudatus	471	0.53
	L	*G. ingrahami*	4429	4.87
		M. prehensilis	3657	3.99
		P. aedium	5374	6
		P. longicaudatus	5734	5.92
Brotomys voratus	H1	*G. ingrahami*	377	1
		M. prehensilis	383	1.1
		P. aedium	387	1.05
		P. longicaudatus	191	0.46
	H	*G. ingrahami*	1038	3.08
		M. prehensilis	990	2.91
		P. aedium	1310	3.9
		P. longicaudatus	551	1.51
	M1	*G. ingrahami*	1595	4.99
		M. prehensilis	1525	4.72
		P. aedium	2211	7.23
		P. longicaudatus	1056	3.16
	M	*G. ingrahami*	1961	6.12
		M. prehensilis	1869	5.62
		P. aedium	2747	8.89
		P. longicaudatus	1490	4.27
	L	*G. ingrahami*	6421	11.31
		M. prehensilis	6313	11.05
		P. aedium	9413	16.86
		P. longicaudatus	8761	12.23
Boromys offella	H1	*G. ingrahami*	325	0.75
		M. prehensilis	238	0.54
		P. aedium	453	1.06
		P. longicaudatus	224	0.48
	H	*G. ingrahami*	835	2
		M. prehensilis	599	1.46
		P. aedium	1236	3.07
		P. longicaudatus	626	1.42
	M1	*G. ingrahami*	1229	3
		M. prehensilis	878	2.16
		P. aedium	1815	4.63
		P. longicaudatus	1043	2.44
	M	*G. ingrahami*	1534	3.63

		M. prehensilis	1097	2.59
		P. aedium	2197	5.35
		P. longicaudatus	1432	3.19
	L	*G. ingrahami*	6025	8.53
		M. prehensilis	5223	7.16
		P. aedium	8387	12.13
		P. longicaudatus	8587	10.56

Table S7. Mapping parameters used in CLC Workbench.

Mapping parameter	Length fraction	Similarity fraction
default	0.5	0.8
L	0.7	0.7
M	0.8	0.8
M1	0.85	0.85
H	0.9	0.9
H1	0.95	0.95
XH	1	1

Table S8. Echimyid outgroup taxa for whole mitochondrial genome and nuclear genes.

Species	GenBank accession number					
	mitogenome	apoB	RBP3	GHR	RAG1	vWF
Callistomys pictus	KU892754	KY303652.1	KY303656.1	KY303654.1	KY303658.1	KJ742614.1
Capromys pilorides	KU892766	KM013977	KM013993	KM013983	JX515322	KM013998
Carterodon sulcidens	KU892752	KY303653	KY303657	KY303655	KJ742678	—
Cavia porcellus	NC_000884.1	AJ399524	AJ427248	AF433930	JN414957	AJ224663
Chinchilla lanigera	NC_021386.1	—	AF297280	AF332036	KF590658.1	AJ238385.1
Clyomys laticeps	KU892753	JX515304	JX515316	JX515326	—	AJ849306
Coendou sp.	NC_021387.1	—	—	—	—	—
Ctenomys sp.	HM544130.1	JN414055.1	JN414816.1	FJ855214.1	JN414961.1	JN415078.1
Dactylomys sp.	KU762015	—	—	KF590681.1	EU313300.1	KF590667.1
Echimys chrysurus	KU892781	JX515301	AJ427247	JX515533	EU313303	AJ251141
Euryzygomatomys spinosus	KU892755	—	JX515315	JX515327	KJ742680	AJ849319
Geocapromys brownii	KU892767	KM013978	—	KJ742644	KJ742692	KJ742621
Geocapromys ingrahami	KU892768	KM013979	KM013995	KM013985	KM013990	KM014000
Hoplomys gymnurus	KU892779	—	—	JN414758	JN633632	JN415080
Isothrix sinnamariensis	KU892785	JX515309	—	KF590685	EU313312	AJ849309
Kannabateomys amblyonyx	KU892775	—	—	—	—	AJ849310.1
Lonchothrix emiliae	KU892786	—	—	—	—	—
Makalata didelphoides	KU892782	JX515303	JF297756	JX515332	EU313314	AJ849311
Mesocapromys melanurus	KU892769	—	—	—	KJ742691	—
Mesomys hispidus	KU892787	JX515305	—	JX515335	EU313322	KF590671
Mesomys stimulax	KU892788	—	—	KJ742630.1	KJ742674.1	KJ742618.1
Myocastor coypus	KU892780	JX515307	JN414814	—	JN414955	AJ251140
Mysateles prehensilis	KU892770	KM013980	—	—	—	—
Octodon lunatus/degus	HM544134.1	—	AM050863	—	KJ742676	AJ238386
Olallamys albicauda	KU892774	—	—	KF590691.1	—	KF590673.1
Pattonomys semivillosus	KU892783	—	—	—	KJ742687.1	KJ742616.1
Phyllomys blainvillii	KU892756	JX515302	JF297781	JX515331	JX515323	AJ849314
Phyllomys dasythrix	KU892757	—	JF297757.1	KJ742641.1	KJ742689.1	JF297709.1
Phyllomys lundi	KU892758	—	JF297770.1	—	—	JF297721.1
Phyllomys mantiqueirensis	KU892759	—	JF297769.1	—	—	JF297720.1
Phyllomys pattoni	KU892760	—	JF297803	KJ742642	KJ742690	JF297754
Plagiodontia aedium	KU892771	KM013981	KM013996	KM013986	KM013991	KM014002
Proechimys cuvieri	KU892778	—	JX515318.1	KF590693.1	KF590665.1	KF590675.1
Proechimys longicaudatus	HM544128.1	—	—	FJ855217	KJ742681	KJ742619
Proechimys roberti	KU892772	—	—	—	—	—
Santamartamys rufodorsalis	KU892777	—	—	—	—	—
Spalacopus cyanus	HM544133.1	—	—	—	—	—
Thrichomys apereoides	KU892773	—	JX515319	JX515325	EU313334	KY303660
Toromys grandis	KU892784	—	—	KF590694.1	EU313336.1	KF590676.1
Trinomys albispinus	KU892761	KM013982	KM013997	KM013987	KM013992	KM014003
Trinomys dimidiatus	JX312694.1	—	—	—	KJ742682.1	KJ742620.1
Trinomys iheringi	KU892762	—	—	KF590695	EU313337	KF590677
Trinomys paratus	KU892763	JX515306	JX515320	JX515330	—	AJ849316
Trinomys setosus	KU892764	—	—	JX515329	—	AJ849317
Trinomys yonenagae	KU892765	KM013982	—	JX515328	—	AJ849318
Tympanoctomys barrerae	HM544132.1	—	—	KX646530.1	—	—

Table S9. Substitution models chosen for partitioned dataset.

Subset	Best model	Subset Partitions
**Mitochondrial genome**		
1	GTR+G	rrnL, rrnS, trnC, trnD, trnF, trnH, trnI, trnK, trnL1, trnL2, trnM, trnN, trnP, trnQ, trnR, trnS2, trnV, trnW, trnY
2	GTR+G	atp6, atp8, cob, cox3, nad1, nad2, nad3, nad4a, nad4l, nad5, nad6
3	GTR+G	cox1, cox2, trnA, trnE, trnG, trnS1, trnT
**apoB** (apolipoprotein B)		
1	HKY+G	Gene
**RAG1** (recombination activating gene 1)		
1	K80+G	Gene
**vWF** (von Willebrand factor)		
1	K80+G	Gene
**RBP3** (interphotoreceptor retinoid binding)		
1	HKY+G	Gene
**GHR** (growth hormone receptor)		
1	K80+G	Gene

Table S10. Fossil constraints and priors used in divergence dating analysis.

Node	Fossil and Deposit Locality	Age (Ma)	Prior distribution
Cavioidea–Erethizontoidea	Minimum: *Andemys termasi* (Tinguirirican) Maximum: *Canaanimys* and *Cachiyacuy* (Barrancan)	31.3–45.9	logNormalPrior mean="5.4" stdev="0.8" offset="31.3"
Octodontidae	Minimum: *Pseudoplateomys innominatus* (Huayquerian) Maximum: *Acarechimys* (Laventan)	6.8–11.8	logNormalPrior mean="1.846" stdev="0.8" offset="6.0"
Trinomys and *Clyomys* + *Euryzygomatomys*	*Theridomysops parvulus* (Huayquerian)	6.0–11.8	logNormalPrior mean="2.141" stdev="0.8" offset="6.0"
Echimyini	*Maruchito trilofodonte* (Colloncuran)	15.7–24.2	logNormalPrior mean="4.15" stdev="0.8" offset="15.7"
Myocastorini	*Pampamys emmonsae* (Huayquerian)	6.0–11.8	logNormalPrior mean="2.141" stdev="0.8" offset="6.0"

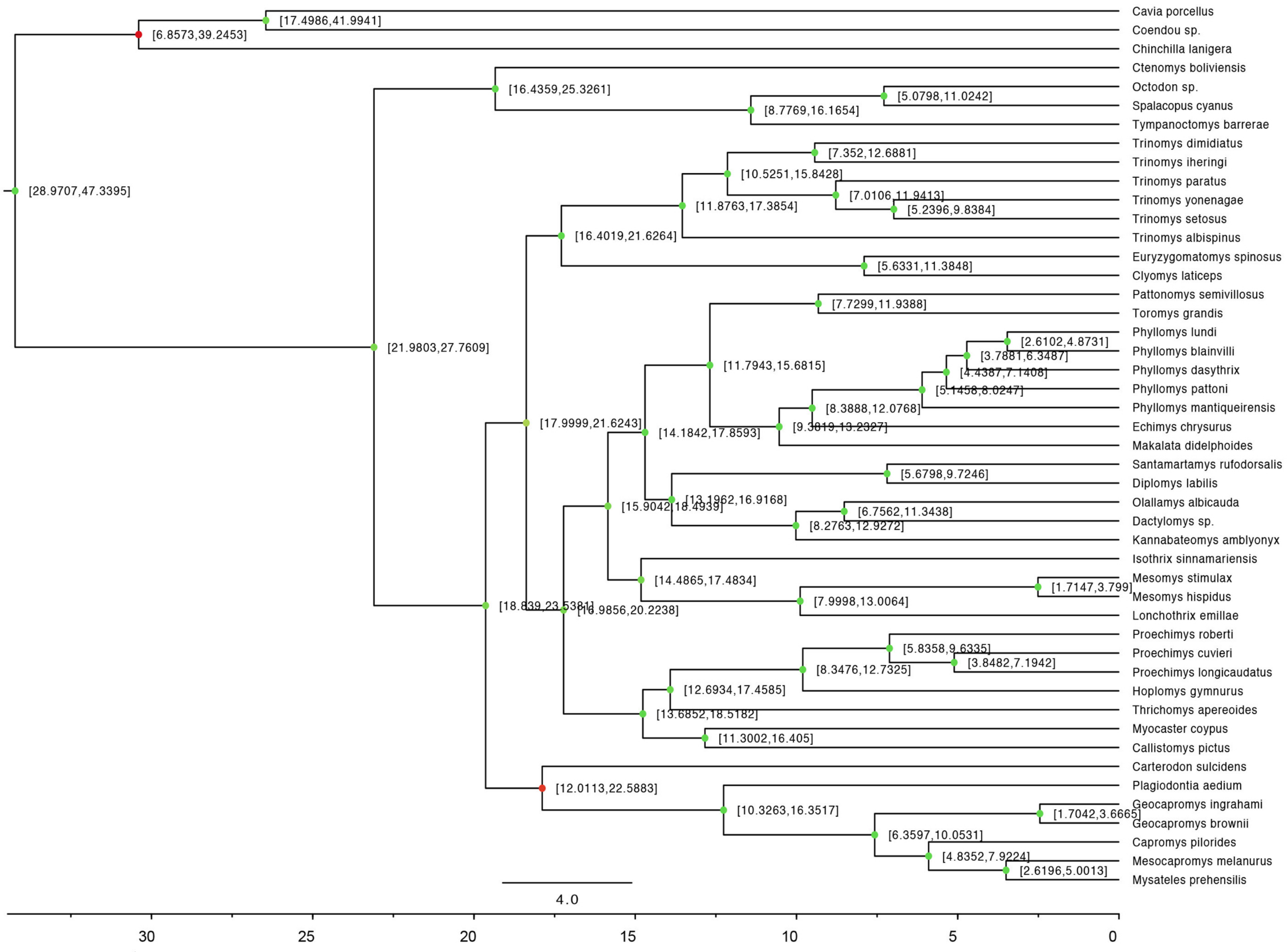

Figure S1. Time-calibrated phylogeny showing estimated divergence dates for Caribbean caviomorphs and mainland Neotropical caviomorphs, excluding extinct taxa. Node colours represent posterior probabilities (red < 1, green = 1). Numbered nodes include 95% error estimates for divergence dates in brackets.

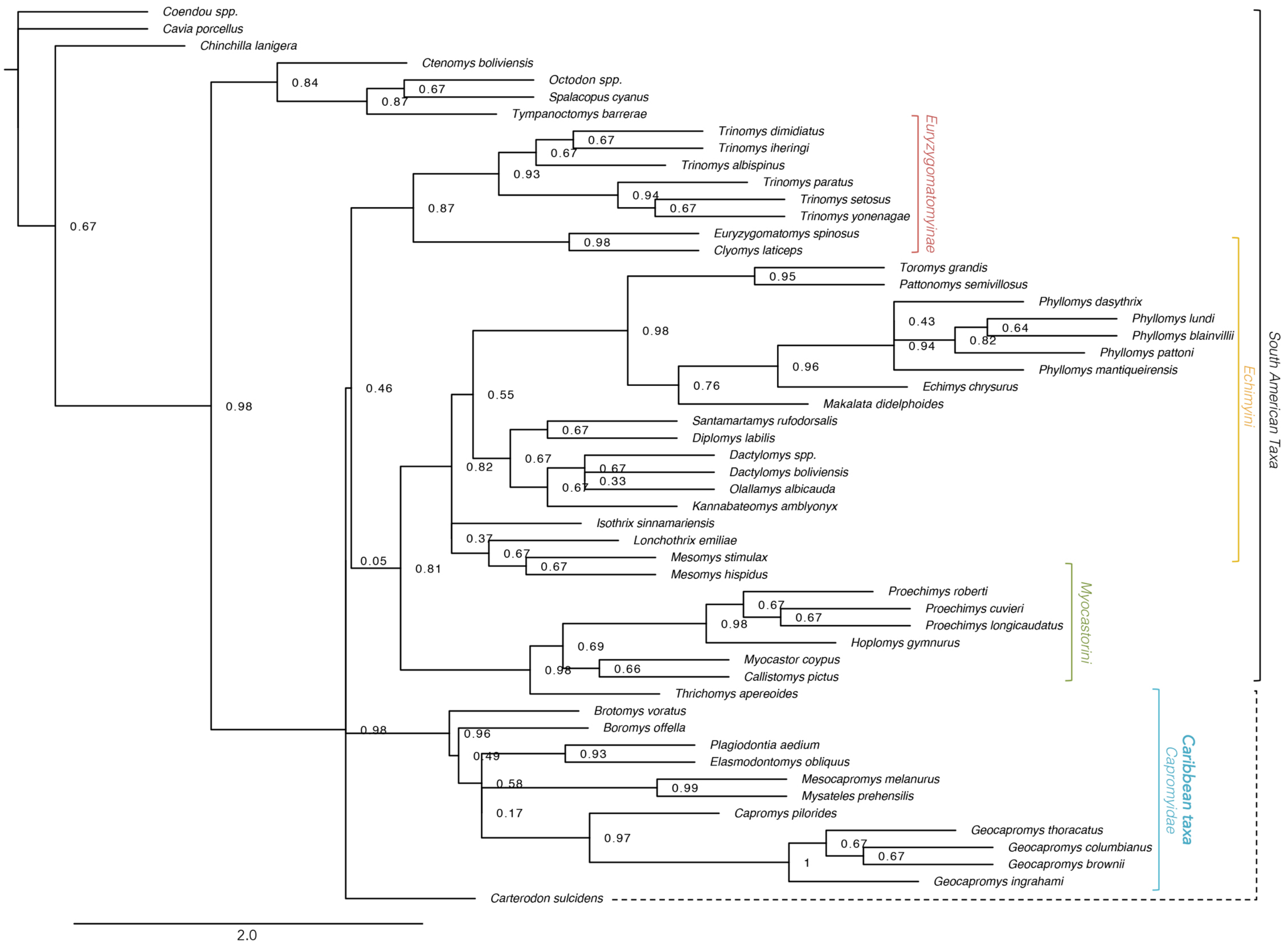

Figure S2. Coalescent species tree inferred using six gene trees. Node values represent the local posterior probability. Normalised quartet score = 0.8. Main clades are colour-coded and named.
